# Associations of Anxiety and Depression With Skin Symptoms and Inflammatory Cytokines in Patients With Atopic Dermatitis

**DOI:** 10.62641/aep.v53i5.1989

**Published:** 2025-10-05

**Authors:** Ting Yang, Jihui Chen, Qin Zou, Siqi Chen, Zhiqiu Lin, Muxiang Yang, Yan Zhang

**Affiliations:** ^1^Department of Dermatology & Venereology, West China Hospital, Sichuan University, 610041 Chengdu, Sichuan, China; ^2^West China School of Nursing, Sichuan University, 610041 Chengdu, Sichuan, China

**Keywords:** anxiety, depression, atopic dermatitis, pruritus, inflammatory cytokine

## Abstract

**Background:**

Atopic dermatitis (AD) is a chronic inflammatory skin disease frequently associated with psychological comorbidities such as anxiety and depression.

**Objective:**

This study aimed to analyse the influence of anxiety and depression on dermatological symptoms and inflammatory cytokines in patients with AD.

**Methods:**

A retrospective cross-sectional analysis was conducted on the clinical records of 241 patients with AD treated at our hospital from December 2022 to December 2024, selected from an initial pool of 320 patients with AD. Sixty-one patients with anxiety/depression comprised the study group, whereas the remaining 180 served as controls. The patients were evaluated using the visual analogue scale (VAS) for pruritus and the Scoring Atopic Dermatitis (SCORAD), and the correlations of anxiety and depression levels with VAS and SCORAD scores were analysed. In addition, inflammatory cytokine levels (interleukin-6 [IL-6] and tumour necrosis factor-α [TNF-α]) were measured in both groups, and their associations with anxiety/depression scores were analysed.

**Results:**

Among the 241 patients with AD, the study identified 19 cases of anxiety, 16 cases of depression and 26 cases of comorbid anxiety and depression. The study group presented significantly higher Hamilton Depression Rating Scale (HAMD), Hamilton Anxiety Rating Scale, Depression Self-Rating Scale for Children (DSRSC) and Screen for Child Anxiety Related Emotional Disorders scores than the control group (*p* < 0.05). The study group had notably higher VAS and SCORAD scores than the control group (*p* < 0.05). Significant moderate positive correlations of anxiety/depression scores with VAS/SCORAD scores were found (*p* < 0.05). All anxiety/depression scores showed significant but generally weak to moderate positive correlations with IL-6 levels (all *p* < 0.05). For TNF-α, only HAMD demonstrated a moderate correlation (r = 0.4228, *p* < 0.0001), and DSRSC showed a weak but significant association (r = 0.2424, *p* = 0.0040).

**Conclusion:**

Anxiety and depression are common among patients with AD, and these emotional states are significantly associated with skin rash and pruritus symptoms and inflammatory responses in patients. Therefore, the psychological well-being of patients should be given due consideration.

## Introduction

Atopic dermatitis (AD) is a chronic inflammatory skin condition [1], with primary clinical manifestations including papules, eczema, oedema, crusting and scaling, often accompanied with hyperpigmentation or hypopigmentation in the healed areas [2]. In recent years, the incidence of AD has gradually increased, bringing a series of adverse effects on patients and the society [3].

The pathogenesis of dermatitis involves multiple interactions, including immune dysregulation from genetic predisposition, environmental triggers and skin barrier dysfunction, which collectively facilitate disease development through a complex immune-neuro-cutaneous network [4, 5]. Notably, psychological status has been recognised as one of the influencing factors in skin disease [6, 7]. For instance, Zhao *et al*. [8] concluded that psychological stress delays dermatitis recovery by exacerbating itch sensitisation in AD. This phenomenon may be explained by AD’s potential to activate the hypothalamic–pituitary–adrenal (HPA) axis, promoting the release of pro-inflammatory cytokines such as interleukin-6 (IL-6) and tumour necrosis factor-α (TNF-α), which can aggravate cutaneous inflammatory responses [9]. Inflammation plays a crucial role in the pathogenesis of AD [10]. Abnormalities in the immune system result in the release of skin inflammatory mediators and create a vicious cycle between pruritus and inflammation, further disrupting skin barrier function and exacerbating the condition [11].

Although the association between AD and psychological factors has been preliminarily established [12], current research examining the influence of anxiety and depression on clinical symptoms and inflammatory cytokines in patients with AD remains limited. Accordingly, this study included clinical data from 241 patients with AD to explore the current status of their anxiety and depression emotions and the impact on their skin symptoms and inflammatory cytokines, thereby providing a reference basis for the comprehensive therapy and prevention of AD. Although previous studies have focused on psychological comorbidities in patients with AD, this study is the first to simultaneously evaluate the impact of anxiety/depressive emotions on clinical symptoms and inflammatory markers in a mixed paediatric–adult population, providing comprehensive evidence for the psychological-skin-immune mechanism.

## Materials and Methods

### Study Population

A retrospective cross-sectional analysis was conducted on the clinical records of 320 patients with AD treated at our hospital from December 2022 to December 2024. Following the inclusion and exclusion criteria outlined below, 241 eligible patients were enrolled.

Inclusion criteria: The patients met the diagnostic criteria for AD [13], including (1) pruritus; (2) typical morphology and location (flexural eczema) or atypical morphology and location with concomitant xerosis; and (3) a chronic or chronically relapsing course. Patients meeting all three criteria were diagnosed with AD. Additional criteria included a disease duration of over 1 year, age ≥8 years, no relevant treatment history in the week prior to enrolment, no history of primary mental disorders, no language or comprehension impairments, completion of anxiety and depression assessments and age 6 years or older.

The exclusion criteria were as follows: patients with concomitant psoriasis, vitiligo or other skin diseases; severe internal medical conditions; or incomplete clinical data were excluded from the study.

The following strategies were applied to handle missing data: cases with >20% missing items in any psychological or clinical scale were excluded; missing cytokine values (<5%) were imputed using multiple imputation by chained equations; and complete-case analysis was performed, as no missing data (0%) were observed in demographic variables.

**Fig. 1.**
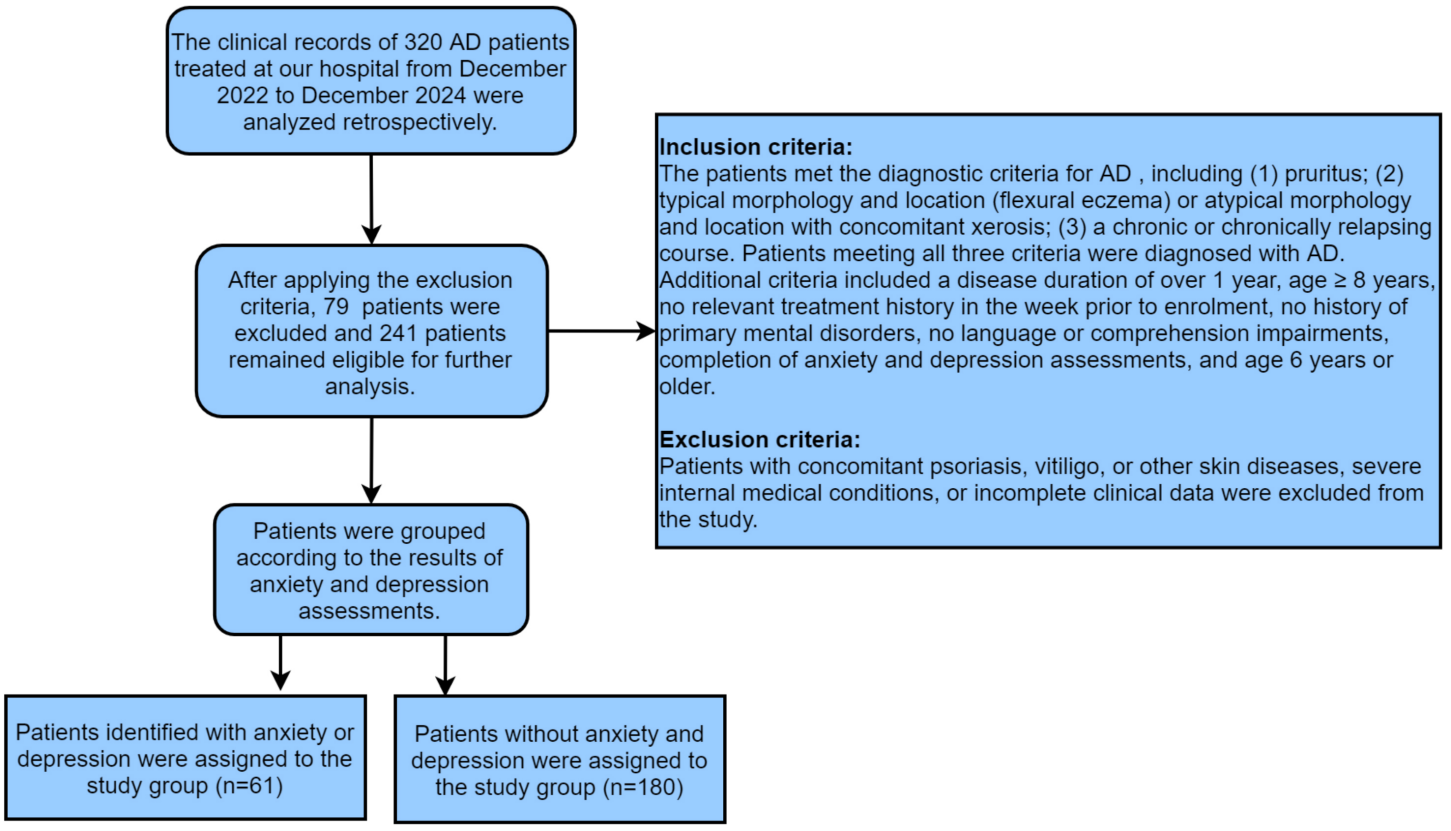
**Screening and grouping process**. Note: AD, Atopic dermatitis.

On the basis of the results of anxiety and depression assessments, 61 patients identified with anxiety or depression were assigned to the study group, and the remaining 180 were assigned to the control group. The screening and grouping process can be found in Fig. 1.

### Data Collection 

#### Clinical and Laboratory Records

The clinical and laboratory records of all patients were collected in January, 2025, including (1) psychological assessments. For patients under 15 years, depression was evaluated using the Depression Self-Rating Scale for Children (DSRSC), whereas anxiety was assessed with the Screen for Child Anxiety Related Emotional Disorders (SCARED). For patients aged 15 and older, clinician-rated scales were used, with the Hamilton Depression Rating Scale (HAMD) measuring depression and Hamilton Anxiety Rating Scale (HAMA) assessing anxiety. All scales were administered face-to-face by trained dermatologists with established inter-rater reliability (κ >0.8). (2) Clinical symptom measures: The visual analogue scale (VAS) scores of pruritus were analysed among patients. The VAS uses a scoring criteria from 0 to 10, with patients rating the severity of their pruritus based on their own perception. Higher scores indicate more severe pruritus [14]. The Scoring Atopic Dermatitis (SCORAD) scores of patients were analysed. The SCORAD involves dimensions such as the extent of skin lesions, severity of skin lesions and subjective symptom scores, with a total score of 0–103 [15]. Higher scores indicate more severe AD. (3) Inflammatory markers. IL-6 and TNF-α in the two groups were analysed. Venous blood (8 mL) was collected from fasting each patient in the morning on the day of their visit. After standing still for 30 min, the blood was treated with 15 min of centrifugation (3000 rpm), followed by collection of the upper layer serum. ELISA was adopted for detecting IL-6 and TNF-α levels under strict instructions provided in the kits for IL-6 (Nanjing Jiancheng Bioengineering Research Institute, A003-1) and TNF-α (Abcam, ab181421). (4) Demographic/clinical characteristics (age, sex, disease duration, lesion location, self-care level and place of residence); with subsequent analyses focusing on correlations between psychological scores (DSRSC/SCARED/HAMD/HAMA) and both clinical symptoms (VAS/SCORAD) and cytokine levels (IL-6/TNF-α), as well as group comparisons based on anxiety/depression status.

#### Psychological Assessment Tools

All psychological scales were administered in Chinese by trained dermatologists, confirming inter-rater reliability (κ >0.8). The DSRSC, a validated Chinese version for adolescents aged 8–14 [16], consists of 18 items (scored 0–2 each; total range 0–36) with a depression cutoff >15 for depression, demonstrating good reliability [Cronbach’s α = 0.82, test–retest intraclass correlation coefficient (ICC) = 0.78]. SCARED (Cronbach’s α = 0.89, test–retest ICC = 0.81), adapted from the Swedish SCARED-R [17], includes 41 items assessing generalised, social and separation anxiety, with a total score >23 indicating anxiety. For participants ≥15 years, clinician-rated HAMD (Cronbach’s α = 0.82, ICC = 0.85) and HAMA (Cronbach’s α = 0.79, ICC = 0.83) scales were used [18, 19], with established cutoffs >17 for depression and >14 for anxiety, respectively. All raters underwent standardised training to ensure consistency.

Although different scales were used for children (SCARED/DSRSC) and adults (HAMA/HAMD), all instruments effectively measured core anxiety/depression constructs using standardised cutoffs and were administered by professional evaluators, indicating high inter-rater reliability (κ >0.8).

### Statistical Analysis

Sample size estimation was performed using GPower 3.1.9.7 (Heinrich-Heine-Universität Düsseldorf, Düsseldorf, Germany). For a two-tailed test with α = 0.05, power (1-β) = 0.95 and anticipated medium effect size (Cohen’s d = 0.5), the required sample size was 210 to detect significant differences in primary outcomes (VAS/SCORAD scores). Our final sample (n = 241) exceeded this threshold, ensuring adequate statistical power.

All statistical analyses were performed using SPSS 20.0 (IBM Corp, Armonk, NY, USA), and graphs were plotted via GraphPad Prism 7 (GraphPad Software, San Diego, CA, USA). Inter-group comparisons of counting data (n [%]) were conducted via the chi-square test, expressed by χ^2^. Normality of the measurement data was assessed using the Shapiro–Wilk test, and all the measurement data were normally distributed and expressed as ($\bar{x}$ ± SD). Between-group and within-group comparisons of measurement data were performed using independent sample *t*-tests and paired *t*-tests, respectively. Correlation was analysed with Pearson’s correlation coefficient. Multivariable linear regression models were constructed to assess independent associations, adjusting for disease duration (continuous) and age group (<15 vs ≥15 years). Psychological scores were analysed as continuous variables after confirming the linearity assumptions by scatterplots. *p* < 0.05 indicates a significant difference.

We assessed potential multicollinearity among key variables (HAMA, HAMD, IL-6 and TNF-α) using variance inflation factors (VIFs) based on a hypothetical linear model including all predictors, although multivariate regression was not part of our primary analysis. All VIFs ranged from 1.21 to 2.31 (**Supplementary Table 1**), well below the threshold of 5, indicating acceptable multicollinearity levels. The correlation matrix between psychological measures (HAMA/HAMD) and inflammatory markers (IL-6/TNF-α) revealed the strongest association between HAMA and HAMD scores (r = 0.62, *p* < 0.001; **Supplementary Table 2**), which remained below a concerning level.

## Results

### Clinical Baseline Data of Patients

An analysis comparing the clinical baseline data of the two groups revealed no significant differences regarding age, sex, ethnicity, disease duration, location of dermatitis, self-care level and place of residence (all *p* > 0.05, Table 1). Although the sex distribution was uneven between the two groups, preliminary analysis showed no significant interaction effect between sex and psychological status on the outcome measures (all *p* > 0.05).

**Table 1.** **Clinical baseline data of patients**.

Factors		Control group (n = 180)	Study group (n = 61)	χ^2^/t	*p*
Average age (years)		23.4 ± 8.32	24.1 ± 7.12	1.521	0.1301
Age				1.064	0.3023
	<15 years	108 (60.0%)	32 (52.5%)		
	≥15 years	72 (40.0%)	29 (47.5%)		
Sex				1.486	0.2228
	Male	81 (45.0%)	22 (36.1%)		
	Female	99 (55.0%)	39 (63.9%)		
Ethnicity				/	0.3850
	Han ethnic	169 (93.9%)	55 (90.2%)		
	Non-Han ethnic	11 (6.1%)	6 (9.8%)		
Course of disease		6.54 ± 2.11	6.72 ± 2.24	1.482	0.1394
Location of dermatitis				2.074	0.3545
	Eyelid	51 (28.3%)	17 (27.9%)		
	Private parts	63 (35.0%)	27 (44.3%)		
	Others	66 (36.7%)	17 (27.9%)		
Self-care level				0.848	0.3571
	Low level	77 (42.8%)	22 (36.1%)		
	Medium-high level	103 (57.2%)	39 (63.9%)		
Place of incidence				0.224	0.6360
	Rural areas	121 (67.2%)	43 (70.5%)		
	Urban areas	59 (32.8%)	18 (29.5%)		

### Detection of Anxiety and Depression

**Fig. 2.**
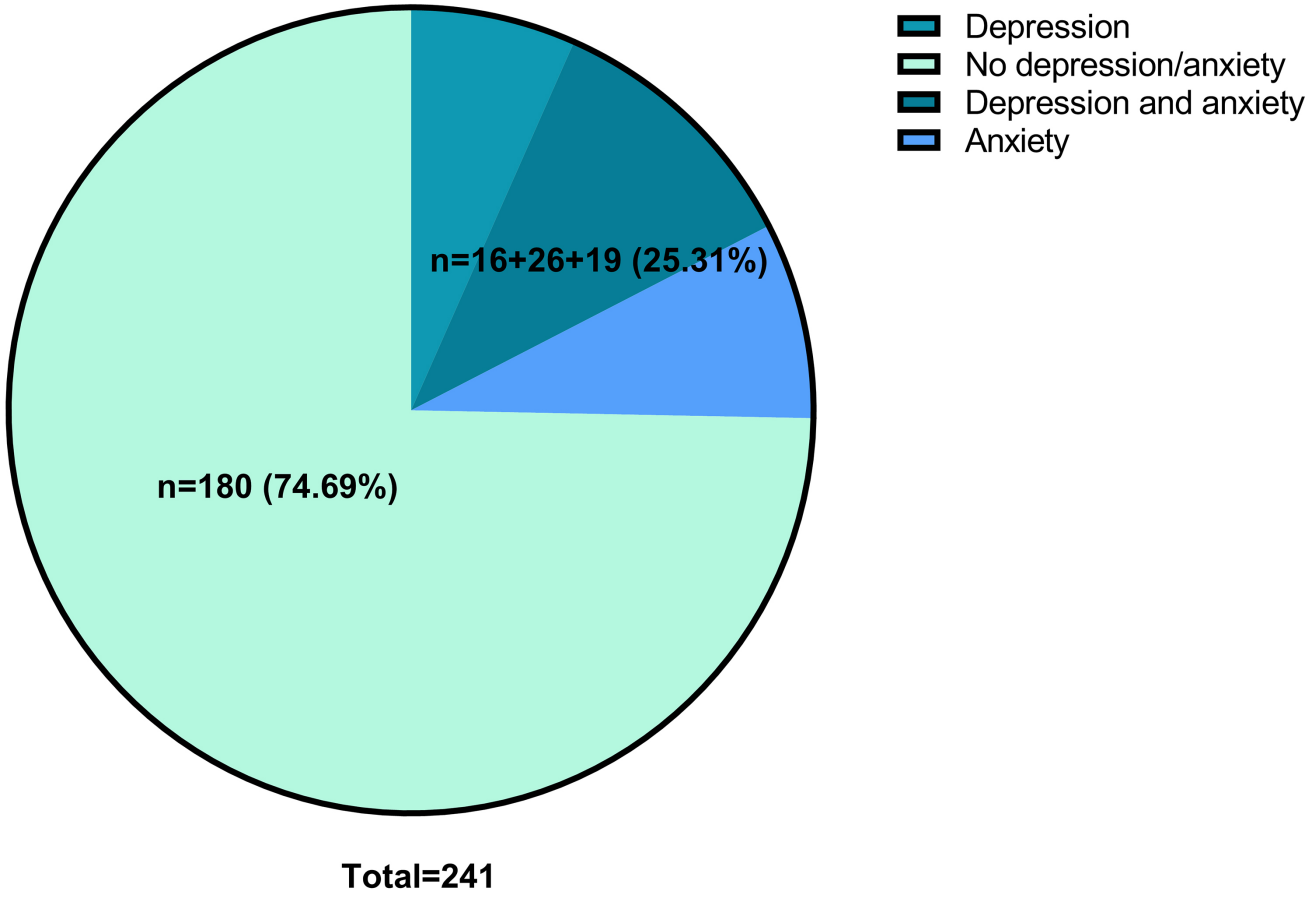
**Detection of anxiety and depression**.

Among 241 patients with AD included, 19 were detected with anxiety, 16 were detected with depression and 26 were detected with comorbid anxiety and depression, resulting in a detection rate of 25.31% (Fig. 2).

### Comparison of Depression and Anxiety

**Fig. 3.**
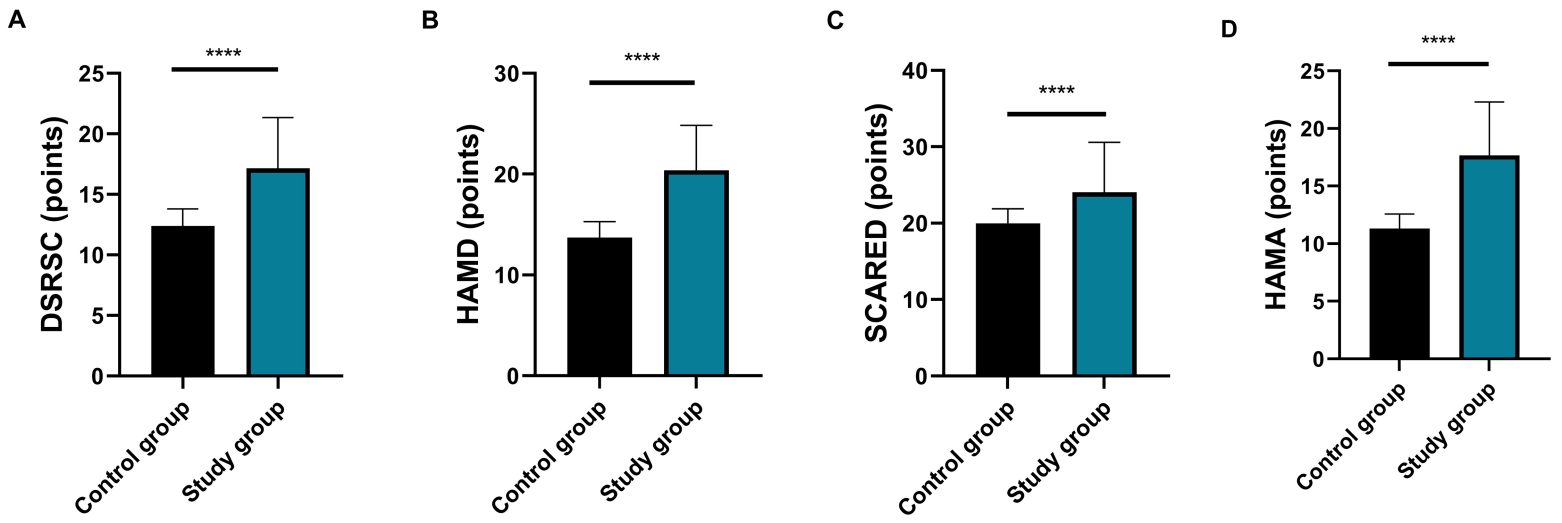
**Comparison of depression and anxiety levels between the two groups**. (A) Comparison of DSRSC scores between the two groups (d = 1.42, 95% CI: 1.08–1.76; ****p < 0.0001); (B) Comparison of HAMD scores between the two groups (d = 1.87, 95% CI: 1.51–2.23; ****p < 0.0001); (C) Comparison of SCARED scores between the two groups (d = 0.95, 95% CI: 0.64–1.26; ****p < 0.0001); (D) Comparison of HAMA scores between the two groups (d = 1.63, 95% CI: 1.28–1.98; ****p < 0.0001). Notes: Control group: n = 180; study group: n = 61. DSRSC, Depression Self-Rating Scale for Children; HAMD, Hamilton Depression Rating Scale; SCARED, Child Anxiety Related Emotional Disorders; HAMA, Hamilton Anxiety Rating Scale. ‘d’ refers to Cohen’s d effect size, interpreted as 0.2 = small, 0.5 = medium and ≥0.8 = large effect. Higher values indicate greater clinical significance of between-group differences.

In the control group, patients under 15 years old had DSRSC and SCARED scores of 12.38 ± 1.40 and 19.99 ± 1.90, respectively. Patients aged 15 and above had HAMD and HAMA scores of 13.72 ± 1.57 and 11.31 ± 1.27, respectively. In the study group, patients under 15 years old had DSRSC and SCARED scores of 17.16 ± 4.19 and 24.06 ± 6.53, respectively. Patients aged 15 and above had HAMD and HAMA scores of 20.40 ± 4.44 and 17.67 ± 4.63, respectively. The study group had notably higher HAMD, HAMA scores or DSRSC and SCARED scores than the control group (all *p* < 0.0001, Fig. 3).

### Comparison of Pruritus Severity 

**Fig. 4.**
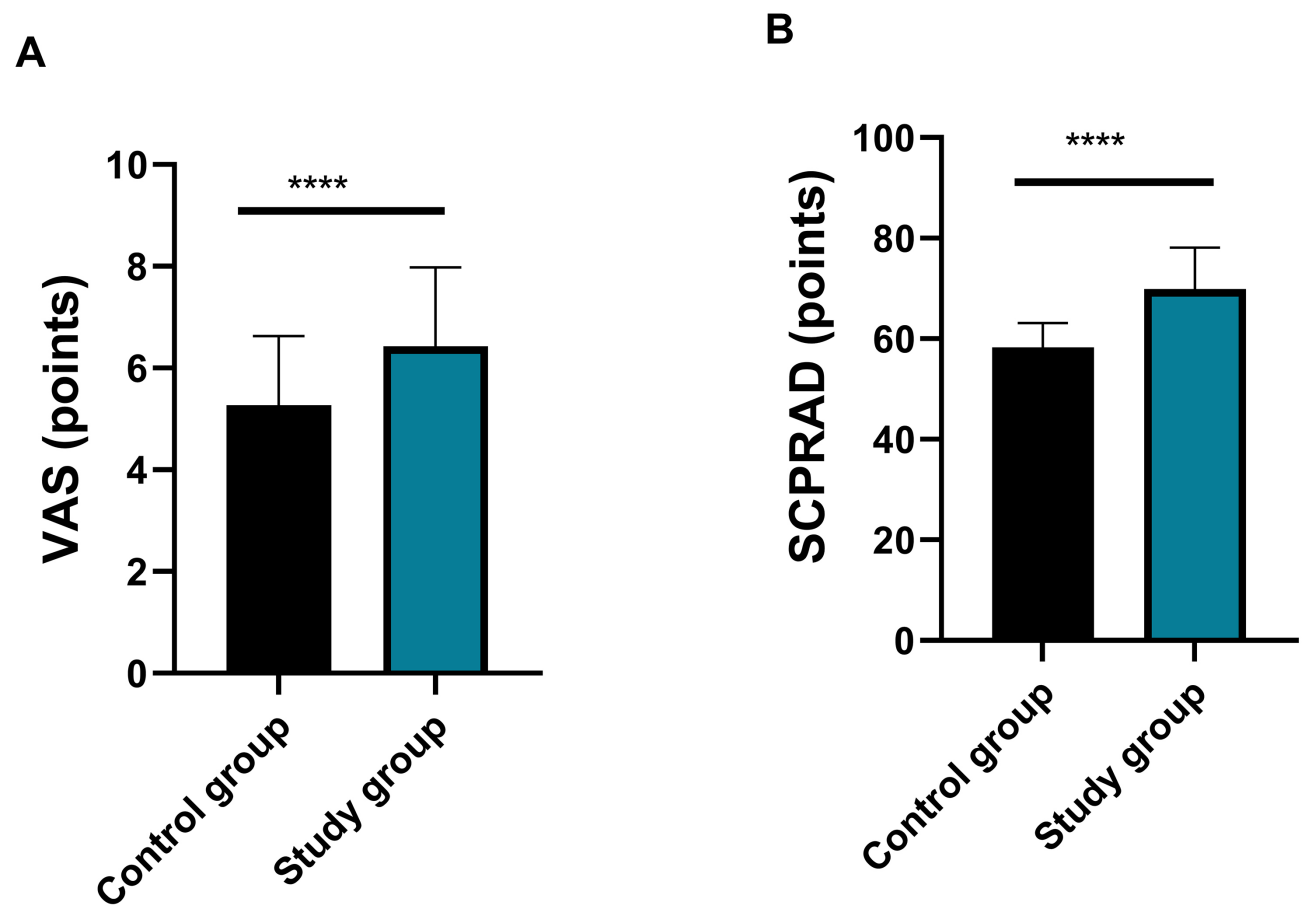
**Comparison of pruritus severity between the two groups**. (A) Comparison of VAS scores between the two groups (d = 1.78, 95% CI: 1.44–2.12; ****p < 0.0001); (B) Comparison of SCORAD scores between the two groups (d = 1.72, 95% CI: 1.38–2.06; ****p < 0.0001). Notes: Control group: n = 180; study group: n = 61. VAS, visual analogue scale; SCORAD, scoring atopic dermatitis. ‘d’ refers to Cohen’s d effect size, interpreted as: 0.2 = small, 0.5 = medium, ≥0.8 = large effect. Higher values indicate greater clinical significance of between-group differences.

The control group had VAS and SCORAD scores of 5.27 ± 1.36 and 58.30 ± 4.79, respectively, whereas the study group had VAS and SCORAD scores of 7.61 ± 1.28 and 69.85 ± 8.24, respectively. The control group had notably lower VAS and SCORAD scores than the study group (both *p* < 0.0001, Fig. 4).

### Comparison of Inflammatory Cytokines

**Fig. 5.**
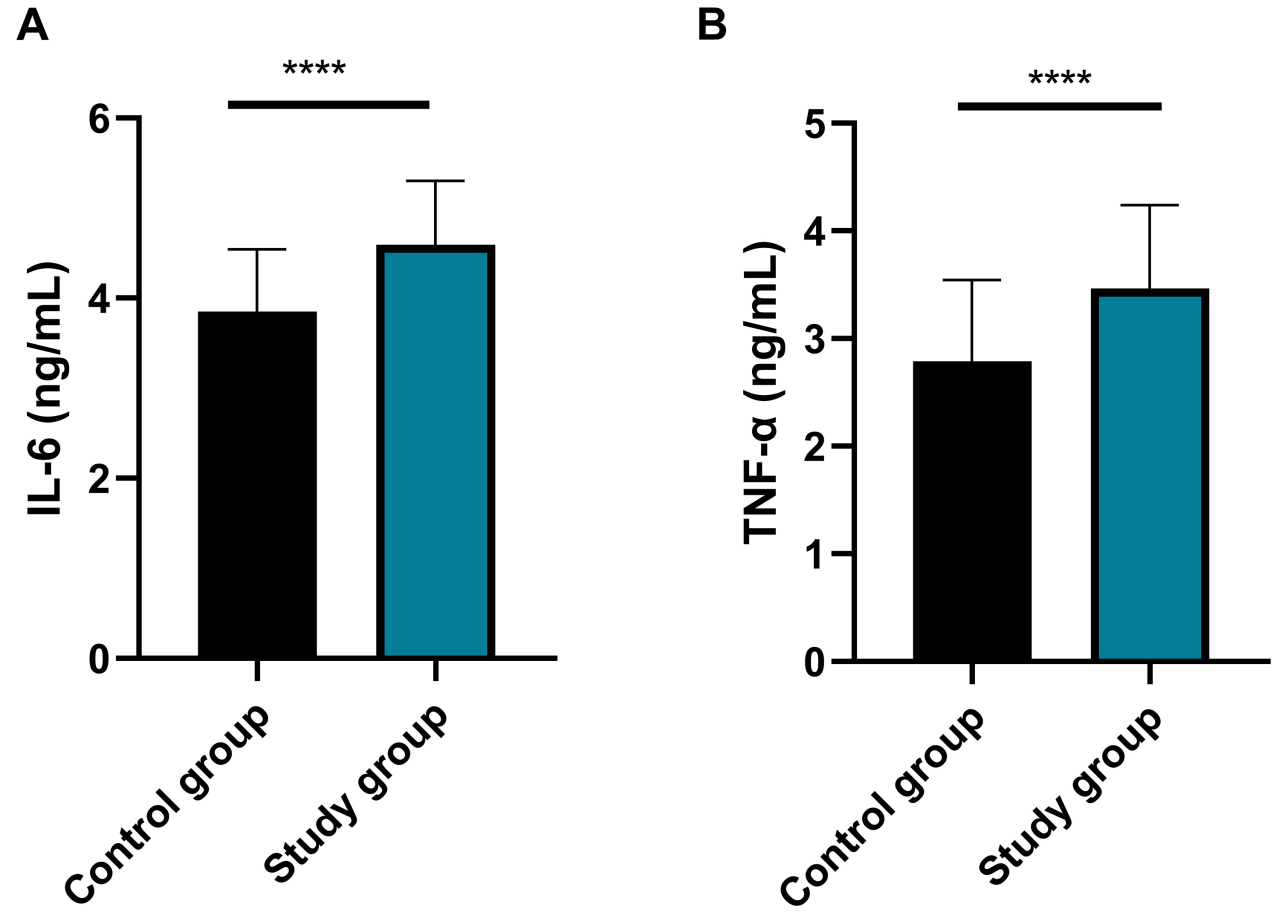
**Comparison of the levels of inflammatory cytokines between the two groups**. (A) Comparison of IL-6 between the two groups (d = 1.07, 95% CI: 0.76–1.38; ****p < 0.0001); (B) Comparison of TNF-α between the two groups (d = 0.88, 95% CI: 0.58–1.18; ****p < 0.0001). Notes: Control group: n = 180; study group: n = 61. IL-6, interleukin-6; TNF-α, tumour necrosis factor-α. ‘d’ refers to Cohen’s d effect size, interpreted as 0.2 = small, 0.5 = medium and ≥0.8 = large effect. Higher values indicate greater clinical significance of between-group differences.

The control group had IL-6 levels of 38.50 ± 6.90 pg/mL and TNF-α levels of 27.90 ± 7.50 pg/mL, and the study group had IL-6 levels of 45.90 ± 7.10 pg/mL and TNF-α levels of 34.60 ± 7.70 pg/mL. The control group showed notably lower IL-6 and TNF-α levels than the study group (both *p* < 0.0001, Fig. 5).

### Relationships of Patients’ Anxiety and Depression With VAS and SCORAD Scores

Significant moderate positive correlations were found between patients’ anxiety and depression scores and VAS and SCORAD scores (all *p* < 0.05, Table 2), with HAMA showing the strongest association with VAS (r = 0.5248).

**Table 2.** **Relationship between patients’ anxiety and depression scores and VAS and SCORAD scores**.

Indexes	VAS score			SCORAD score		
	r	*R* ^2^	*p*	r	*R* ^2^	*p*
DSRSC	0.3079	0.096***	0.0002	0.4472	0.203****	<0.0001
SCARED	0.3250	0.109****	<0.0001	0.2879	0.084***	0.0006
HAMD	0.4089	0.168****	<0.0001	0.4228	0.176****	<0.0001
HAMA	0.5248	0.270****	<0.0001	0.4322	0.185****	<0.0001

Notes: ***p < 0.001, ****p < 0.0001; VAS, visual analogue scale; SCORAD, Scoring Atopic Dermatitis; DSRSC, Depression Self-Rating Scale for Children; SCARED, Child Anxiety-related Emotional Disorders; HAMD, Hamilton Depression Rating Scale; HAMA, Hamilton Anxiety Rating Scale.

### Relationships Between Patients’ Anxiety and Depression and IL-6 and TNF-α Levels

As shown in Table 3, except for non-significant associations between SCARED/HAMA scores and TNF-α (both *p* > 0.05), anxiety/depression scores showed significant but generally weak to moderate correlations with IL-6 levels (r range: 0.1949–0.4144, all *p* < 0.05), whereas only HAMD demonstrated moderate correlations with TNF-α (r = 0.4228, *p* < 0.0001).

**Table 3.** **Relationships between patients’ anxiety and depression scores and IL-6 and TNF-α levels**.

Indexes	IL-6			TNF-α		
	r	*R* ^2^	*p*	r	*R* ^2^	*p*
DSRSC	0.1949	0.036*	0.0215	0.2424	0.058**	0.0040
SCARED	0.3038	0.090**	0.0003	0.1462	ns	0.0860
HAMD	0.4144	0.168****	<0.0001	0.4228	0.176****	<0.0001
HAMA	0.2887	0.084**	0.0033	0.1647	ns	0.0980

Notes: *p < 0.05, **p < 0.01, ****p < 0.0001; ns = non-significant (*p* > 0.05). IL-6, interleukin-6; TNF-α, tumour necrosis factor-α; DSRSC, Depression Self-Rating Scale for Children; SCARED, Child Anxiety-related Emotional Disorders; HAMD, Hamilton Depression Rating Scale; HAMA, Hamilton Anxiety Rating Scale.

### Adjusted Regression for Psychological Factors and AD Outcomes

Separate models were constructed for each outcome. The VAS and IL-6 models were adjusted for disease duration, whereas the SCORAD model was adjusted for age group, based on a priori clinical relevance. After adjustment, each 1-point increase in anxiety score was associated with a 0.12-point increase in VAS (95% CI: 0.08–0.16, *p* < 0.0001) and a 0.3 pg/mL rise in IL-6 levels (95% CI: 0.10–0.50, *p* = 0.0040). Similarly, each 1-point increase in depression score predicted 0.65-point increase in SCORAD (95% CI: 0.51–0.79, *p* < 0.0001), whereas an older age (≥15 years) was associated with a 3.21-point higher SCORAD (*p* < 0.0001; Table 4).

**Table 4.** **Multivariable linear regression analysis of factors associated with VAS, SCORAD and IL-6 levels**.

Dependent Variable	Predictor Variable	β (95% CI)	*p*	Adjusted *R*^2^
VAS	HAMA score	0.12 (0.08, 0.16)	<0.0001****	0.41
	Disease duration (years)	0.08 (0.02, 0.14)	0.0130*	
SCORAD	HAMD score	0.65 (0.51, 0.79)	<0.0001****	0.38
	Age ≥15 years	3.21 (1.85, 4.57)	<0.0001****	
IL-6	HAMA score	0.30 (0.10, 0.50)	0.0040**	0.29
	Disease duration (years)	0.20 (0.00^†^, 0.40)	0.0420*	

Notes: VAS, visual analogue scale; SCORAD, Scoring Atopic Dermatitis; IL-6, interleukin-6. All models were run separately with the most clinically relevant covariate: VAS and IL-6 models adjusted for disease duration; SCORAD model adjusted for age group. ^†^: The lower limit of the CI is >0 but rounded to ‘0.00’, indicating statistical significance (CI excludes zero). *p < 0.05. **p < 0.01, ****p < 0.0001.

## Discussion

AD is a persistent, recurrent disorder often unresponsive to definitive treatment [20, 21]. Patients frequently experience sleep disturbances, emotional dysregulation and compulsive scratching behaviours [22]. Chronic anxiety and depression may worsen symptoms and trigger recurrence [23], with research confirming significant psychosocial influences on disease progression [24].

Among the 241 patients in this study, there were 19 cases of anxiety, 16 cases of depression and 26 cases where patients experienced anxiety and depression simultaneously, with anxiety combined with depression being predominant. In one study by Vinh *et al*. [25], the proportion of adult patients with AD and mixed anxiety and depression disorders was 1.44%, which differed from the results of this study. In our adult subgroup (n = 101), we observed much higher rates of comorbid anxiety/depression (13.9%) compared with 1.44%. This discrepancy may reflect (1) our inclusion of hospitalised severe cases (mean SCORAD 62.3 vs their 48.6); (2) use of clinician-rated versus self-report measures; and (3) cultural differences in symptom reporting.

This study revealed notably higher VAS and SCORAD scores in the study group compared with the control group, as well as significantly positive associations of the anxiety and depression scores with VAS scores and SCORAD scores. These results suggested that anxiety and depression emotions in patients with AD may exacerbate the symptoms of dermatitis and pruritus. This finding was consistent with the conclusion of a study by Lönndahl *et al*. [26], who reported that chronic stress tends to worsen AD. Furthermore, the research by Zhang* et al*. [7] supported this conclusion, stating that psychological stress is a key factor in the development of many skin diseases and can aggravate dermatological conditions, significantly affecting patients’ quality of life. The causes may include various factors such as an imbalance in neuroimmune regulation, excessive release of inflammatory mediators, changes in self-care behaviours, long-term chronic stress and neuro-skin interactions [25]. Psychological stressors can activate the HPA axis, promote the secretion of stress hormones such as cortisol and angiotensin and affect skin barrier function. For example, stress hormones can reduce epidermal structural proteins and lipids and decrease hydration of the stratum corneum, compromising the body’s skin barrier function [25]. A decrease in skin barrier function can diminish skin antimicrobial and anti-inflammatory functions and stratum corneum cohesion, exacerbating the symptoms of dermatitis and pruritus [27]. The psychological stress induced by anxiety and depression may promote the release of cortisol and catecholamines, both of which affect the body’s immune function. For example, they can promote the differentiation of Th cells into Th2 cells, affecting the function of Th1 cells, thereby exacerbating the degree of allergic inflammatory reactions and leading to dermatitis and pruritus symptoms [28].

This study further revealed close associations between anxiety and depression emotions and the IL-6 and TNF-α levels. The study group presented notably higher IL-6 and TNF-α levels than the control group. Except for the non-significant associations of SCARED and HAMA scores with TNF-α, all anxiety/depression scores showed significant positive correlations with IL-6 levels. This result suggested that anxiety and depression emotions may have a bidirectional relationship with increased levels of inflammatory cytokines in patients. Emotional disorders may influence the release of inflammatory mediators via the neuroimmune regulation system, whereas the increase in inflammatory cytokines may further aggravate emotional disorders, creating a vicious cycle [29]. From a mechanistic perspective, the increase in IL-6 and TNF-α, as key pro-inflammatory cytokines, not only reflects the deterioration of skin barrier integrity but also the dysregulation of skin immune function and intensification of pruritus symptoms [30]. Specifically, IL-6 and TNF-α can intensify the inflammatory response and symptom severity of AD by activating keratinocytes, promoting Th2 immune responses and increasing the release of neuropeptides. Furthermore, emotional states may influence the secretion of inflammatory mediators via the activation of the HPA axis and sympathetic nervous system, thereby aggravating the degenerative procession of AD [31]. The psychological stress associated with anxiety and depression appears to influence AD inflammation through three well-characterised, interacting pathways: (1) HPA axis dysregulation: chronic stress diminishes cortisol secretion while increasing corticotropin-releasing hormone (CRH) production, resulting in mast cell degranulation and IL-6 release via reduced glucocorticoid receptor signalling [32]. (2) Sympathetic overactivation: norepinephrine from sustained β-adrenergic stimulation induces keratinocyte-derived TNF-α via NF-κB activation [33]. (3) Neuropeptide release: substance P from sensory neurons upregulates IL-6 production in dermal fibroblasts through NK-1 receptor binding [34]. This tripartite mechanism elucidates our observed cytokine patterns, with IL-6 showing strong psychogenic modulation due to its dual induction pathways, whereas TNF-α production requires robust neuroimmune stimulation. Moreover, the weaker association between psychological factors and TNF-α (compared with IL-6) suggested that TNF-α may participate in AD progression through distinct neuroimmune pathways, such as Th1/Th17 polarisation. The high threshold for modulation by childhood anxiety (SCARED) or adult depression (HAMD) scores requires verification in large cohorts. Additionally, diurnal fluctuations in TNF-α levels or variations in sampling time points may contribute to these observations. Although we found significant correlations between psychological factors and clinical/inflammatory measures, the proportion of variance explained was limited (*R*^2^ range: 0.036–0.270), which is consistent with established psychodermatological evidence reported by Tsintsadze *et al*. [35]. The corresponding correlation coefficients (r range: 0.1949–0.5248) indicated small to moderate effect strengths. This discrepancy between statistical significance and explained variance likely reflects (1) potential nonlinear psychobiological relationships not captured by linear analyses and (2) measurement variability in psychological assessments and cytokine assays. Despite the modest proportion of variance explained, these associations remain clinically relevant as small effects can be meaningful in chronic multifactorial diseases.

Our study demonstrated that anxiety and depression significantly exacerbated clinical symptoms and inflammatory responses in patients with AD. These findings highlighted the necessity of integrating mental health care into standard dermatological practice. Prior studies indicated that cognitive behavioural therapy for dermatology (CBT-D) significantly reduces pruritus severity and IL-6 in patients with AD [36], and mindfulness-based stress reduction (MBSR) improves skin symptoms by modulating stress responses [37]. Therefore, we recommend the routine implementation of CBT-D and MBSR in AD management protocols, particularly for patients with SCORAD >60 or elevated inflammatory markers.

This study has several important limitations that warrant consideration. The single-centre, retrospective design with a homogeneous Han Chinese cohort (n = 241) may limit generalisability, and the cross-sectional nature prevents establishing causal relationships between psychological factors and AD severity. Additionally, potential residual confounding and measurement variability from using age-stratified scales may influence the results. Notably, the lack of sensitivity analyses to evaluate potential confounding by unmeasured variables (e.g., socioeconomic status and concurrent therapies) may affect the robustness of the results. Despite these limit ations, our findings highlighted significant psychodermatological associations that merit further investigation through multi-centre prospective studies with diverse populations. Future research should integrate neural imaging (MRI/EEG) with immune profiling to elucidate brain–skin axis mechanisms and evaluate whether psychological interventions like CBT can effectively disrupt the cytokine–symptom cycle and improve clinical outcomes.

## Conclusions

Anxiety and depression emotions are common among patients with AD and are significantly associated with the severity of skin symptoms and elevated levels of inflammatory cytokines. Therefore, in the clinical management of AD, the psychological well-being of patients must be prioritised. Psychological interventions should be considered as an essential component of comprehensive treatment to improve patients’ quality of life and disease prognosis.

## Availability of Data and Materials

All experimental data included in this study can be obtained by contacting the corresponding author if needed.

## Supplementary Material

Supplementary material associated with this article can be found, in the online version, at https://doi.org/10.62641/aep.v53i5.1989.
