# Supplementary material for: Associations of Anxiety and Depression With Skin Symptoms and Inflammatory Cytokines in Patients With Atopic Dermatitis
Source: Actas Esp Psiquiatr. 2025 Oct 5;53(5):999–1010. doi: 10.62641/aep.v53i5.1989 (PMC12538605; doi:10.62641/aep.v53i5.1989)
Supplement: Supplementary file 1 [file ActEsp-53-5-999-1010-s1.docx]

**Supplementary Table 1: Results of variance inflation factor analysis.**

| **Predictor variable** | **VIF** | **1/VIF (Tolerance)** |
| --- | --- | --- |
| HAMA | 2.12 | 0.48 |
| HAMD | 2.31 | 0.43 |
| IL-6 | 1.78 | 0.56 |
| TNF-α | 1.61 | 0.63 |
| Course duration（year） | 1.21 | 0.83 |
| Age group | 1.41 | 0.71 |

Notes: HAMA: Hamilton Anxiety Rating Scale; HAMD: Hamilton Depression Rating Scale; IL-6: interleukin-6; TNF-α: tumour necrosis factor-α.

**Supplementary Table 2: Correlation matrix between psychological measures (HAMA/HAMD) and inflammatory markers (IL-6/TNF-α).**

|  | **HAMA** | **HAMD** | **IL-6** | **TNF-α** | **Disease duration** | **Age grouping** |
| --- | --- | --- | --- | --- | --- | --- |
| HAMA | 1.00 | 0.62* | 0.31* | 0.18 | 0.09 | 0.12 |
| HAMD | 0.62* | 1.00 | 0.28* | 0.15 | 0.11 | 0.08 |
| IL-6 | 0.31* | 0.28* | 1.00 | 0.43* | 0.22* | 0.19 |
| TNF-α | 0.18 | 0.15 | 0.43* | 1.00 | 0.17 | 0.14 |
| Disease duration | 0.09 | 0.11 | 0.22* | 0.17 | 1.00 | 0.05 |
| Age grouping | 0.12 | 0.08 | 0.19 | 0.14 | 0.05 | 1.00 |

Notes: * *p*<0.05; Age group was coded as a binary variable (<15 years old=0, ≥15 years old=1) for the purpose of calculating point-biserial correlation coefficients. HAMA: Hamilton Anxiety Rating Scale; HAMD: Hamilton Depression Rating Scale; IL-6: interleukin-6; TNF-α: tumour necrosis factor-α.
